# Striatal signaling: two decades of progress

**DOI:** 10.3389/fnana.2012.00043

**Published:** 2012-10-16

**Authors:** Emmanuel Valjent

**Affiliations:** ^1^Institut de Génomique FonctionnelleMontpellier, France; ^2^Inserm U661Montpellier, France; ^3^CNRS UMR 5203Montpellier, France; ^4^Université Montpellier I & IIFrance

**Figure 1:**
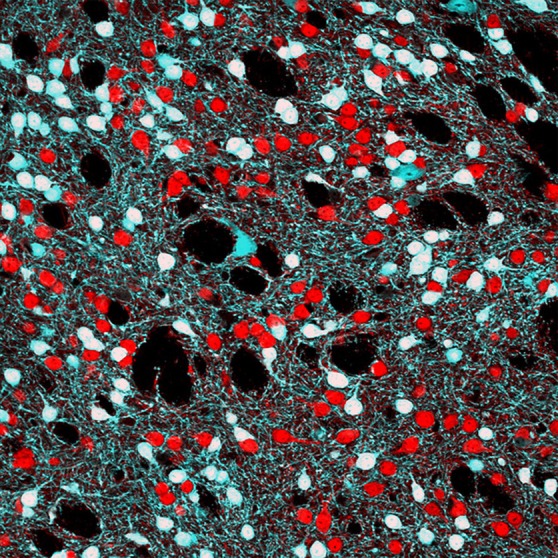
**Single confocal image showing DARPP-32 immunoreactivity (red) and EGFP fluorescence (cyan) in *Drd2-EGFP* BAC transgenic mice.** DARPP-32 allows the identification of all striatal medium-sized spiny neurons (MSNs) while EGFP labels striatopallidal MSNs and large aspiny cholinergic interneurons. Picture by E. Valjent.

The striatum and its ventral extension the nucleus accumbens (NAcc) are the main inputs structure of the basal ganglia circuit (Figure 1). While the striatum appears to be essential for the selection and initiation of actions and for the learning of habits and skills, the NAcc seems to be more involved in motivation and reward. Interestingly, dysfunctional plasticity of these structures has been associated with prominent neurological and psychiatric disorders such as Parkinson's disease, obsessive-compulsive disorder, Tourette's syndrome, and drug addiction.

The aims of this Special Topic was to propose a series of reviews covering our recent understanding on striatal signaling pathways, which are activated by a variety of therapeutic agents or drugs of abuse in physiological and pathological context. The 17 articles provide a deep overview of our current knowledge and highlight how recent advances in cell-type specific technologies, including fluorescent reporter mice, conditional knockout, and viral-mediated gene transfer allow to untangle the complexity of studying signal transduction in the brain *in vivo*. The content of each of these articles is briefly summarized below.

The first article, by Nishi et al. (2011) summarizes some of the mechanisms involved in the modulation of dopamine D1R signaling. The authors review both the canonical and non-canonical D1R signaling cascade that coupled to Gαolf/adenylyl cyclase/PKA signaling and to Gq/phospholipase C or Src family kinase, respectively. They also extensively discuss the role played by Phosphodiesterases (PDEs) in the control of striatal signaling. In the second article, Hervé (2011) reviews the critical role of the highly specialized G-protein subunit heterotrimer containing Gαolf/β2/γ7 that couples D1R and A2aR to adenylyl cyclase 5. The physiological consequences of the alterations of Gαolf levels and/or activity are also extensively discussed. The third article by Perreault et al. (2011) reviews the current knowledge on D1R-D2R heteromers. Although, it is commonly accepted that D1R and D2R are preferentially expressed in striatonigral and striatopallidal MSNs, respectively, the authors put forward the hypothesis that MSNs co-expressing both receptors display unique signaling properties supporting the existence of a “novel third pathway”. Ferré et al. (2011) focus on the role of the different subpopulation of striatal adenosine A2aR. Post-synaptically, A2aR are localized in striatopallidal MSNs where they form heteromers with dopamine D2R, cannabinoid CB1R, and mGluR5R. The authors also highlight how presynaptic A2aR-A1R heteromers can impact on striatal signaling. After a brief overview of the major G-protein-coupled receptors (GPCRs) and their downstream effectors, Xie and Martemyanov (2011) discuss the progress made in understanding the roles of RGS proteins in controlling striatal G-protein signaling. A particular attention has been paid on the role of the striatal-enriched RGS9-2 protein on dopamine and opioid signaling. The sixth article by Del'Guidice et al. (2011) deals with the role of β-arrestin 2 on the regulation of dopamine receptor desensitization as well as the involvement of this scaffoling protein in the control of the Akt/GSK3 signaling pathways. Potential molecular targets of β-arrestin 2/Akt/GSK3 signaling are also reviewed. After an outline of the current knowledge of the signaling cascades that target the nucleus, Matamales and Girault (2011) describe how through the regulation of protein kinases, phosphatases, and transport through the nuclear pore, a signal initiated at the plasma membrane is amplified in the cytosol and then relayed to the nucleus. Identified mechanisms involved in transcription regulation and chromatin re-modeling in MSNs are also discussed. In the eighth article Walaas et al. (2011) provide a comprehensive review on the regulation and roles of the three major subclasses of serine/threonine protein phosphatases, PP1, PP2B, and PP2A. Their direct or indirect regulation by the striatum-enriched phosphoproteins DARPP32, RCS, and ARPP-16 is extensively discussed. The ninth article by Fitzpatrick and Lombroso (2011) also deals with the role of protein phosphatases focusing more specifically on the striatal-enriched protein tyrosine phosphatase (STEP). The structure and the regulation of STEP phosphorylation are described, as are the functional consequences of STEP dysfunctions in various neurological and neuropsychiatric disorders.

The dysregulation of striatal signaling induced by the drugs of abuse leads to an abnormal neuronal plasticity that under normal circumstances serves to shape appropriate reward-related behaviors. After a rapid update on the role played by dopamine receptors in drug addiction, Philibin et al. (2011) perform an in-depth review of intracellular signaling cascades, involving specific kinases and phosphatases affected by cAMP and Ca^2+^ that modulate neuroplasticity to affect behavioral outcome. Particular emphasis is placed on PKA, cdk5, ERK, CamKII, and PKC. Glutamate receptors cooperate closely with dopamine to regulate striatal signaling. In the article of Mao et al. (2011) several major types of post-translational modifications of glutamate receptors, including phosphorylation, palmitoylation, ubiquitination, and sumoylation are discussed. The impact of such modifications on striatal signaling and drug-induced neuronal plasticity is also addressed. The article by Lobo and Nestler (2011) discuss extensively how recent advances in cell-type-specific technologies have advanced the field toward a more comprehensive understanding of the distinct molecular and functional contributions of striatonigral and striatopallidal MSNs in drug addiction. The article by Durieux et al. (2011) also deals with the development of new models and techniques allowing the selective targeting of striatal MSNs and interneurons. Although these approaches represent invaluable tools, the authors point out the need of an extensive characterization to avoid overstated conclusions.

Altered striatal signaling in neurological disease has been also covered in this Special Issue. Thus, the contribution of Murer and Moratalla (2011) deals with the abnormal striatal signaling observed in response to L-DOPA in the context of Parkinson's disease. Transcriptional and translational molecular mechanisms that may translate enhanced D1 signaling into dyskinetic movements are discussed, as are the role of D2/D3R-mediated signaling. Bonito-Oliva et al. (2011) review the key role played by DARPP-32 in the actions of antiparkisonian and antipsychotic drugs. The emerging picture indicating that L-DOPA-induced dyskinesia and extrapyramidal syndrome are linked to abnormal cAMP signaling in striatonigral and striatopallidal MSNs is also discussed. After a brief overview of the clinical aspects, genetics, and neuropathology, Roze et al. (2011) summarize our current knowledge on the molecular mechanisms underlying Huntington's disease. Changes in axonal transport and synaptic dysfunction have been discussed, as are transcriptional dysregulation observed at the striatal level. Finally, the last article by Crittenden and Graybiel (2011) provides a comprehensive review on the current state of knowledge of the details of striosome-matrix compartimentalization on the striatum and alterations in various disease states.

I am grateful for the enthusiasm and willingness shown by so many colleagues in the field of striatal signaling to contribute an article in this Special Issue. Some of the contributors also served as expert reviewers for articles. In addition, I would like to thank external reviewers for their valuable criticisms and suggestions that contributed to the success of this Special Issue.
